# Supplementary material for: End Sequence Analysis Toolkit (ESAT) expands the extractable information from single-cell RNA-seq data
Source: Genome Res. 2016 Oct;26(10):1397–410. doi: 10.1101/gr.207902.116 (PMC5052061; doi:10.1101/gr.207902.116)
Supplement: Supplemental Material [file supp_gr.207902.116_Supp_Materials.tar.gz › ESAT-esat_v0.1/doc/html/classbroad_1_1pda_1_1differential_expression_1_1_permutations.html]

ESAT: broad.pda.differentialExpression.Permutations Class Reference


ESAT


- Main Page
- Related Pages
- Packages
- Classes
- Files

- Class List
- Class Index
- Class Hierarchy
- Class Members

All Classes Namespaces Files Functions Variables Enumerator Pages

- broad
- pda
- differentialExpression
- Permutations

Static Public Member Functions |
List of all members

broad.pda.differentialExpression.Permutations Class Reference

Collaboration diagram for broad.pda.differentialExpression.Permutations:

[legend]

|  |  |
| --- | --- |
| Static Public Member Functions | |
| static MatrixWithHeaders[] | permuteData (MatrixWithHeaders data, Collection< String > group1, Collection< String > group2, int numberPermutations, double[] fudgeFactors, boolean useFold, boolean paired) |
|  | |
| static MatrixWithHeaders[] | permuteData (MatrixWithHeaders data, Map< String, Collection< String >> groups, int numberPermutations, double[] fudgeFactors, boolean useFold, boolean paired) |
|  | |
| static MatrixWithHeaders[] | permuteDataByGroupSize (MatrixWithHeaders data, int groupSize, Map< String, Collection< String >> groups, int numPerm, double[] fudgeFactors, boolean useFold) |
|  | |
| static MatrixWithHeaders[] | permuteAndAssignFDR (MatrixWithHeaders testStats, MatrixWithHeaders data, Collection< String > group1, Collection< String > group2, int numberPermutations, double[] fudgeFactors, boolean useFold, boolean paired, double alpha) |
|  | |

## Member Function Documentation

|  |  |  |  |
| --- | --- | --- | --- |
| static MatrixWithHeaders [] broad.pda.differentialExpression.Permutations.permuteAndAssignFDR | ( | MatrixWithHeaders | *testStats*, |
|  |  | MatrixWithHeaders | *data*, |
|  |  | Collection< String > | *group1*, |
|  |  | Collection< String > | *group2*, |
|  |  | int | *numberPermutations*, |
|  |  | double[] | *fudgeFactors*, |
|  |  | boolean | *useFold*, |
|  |  | boolean | *paired*, |
|  |  | double | *alpha* |
|  | ) |  |  |

static

Here is the call graph for this function:

|  |  |  |  |
| --- | --- | --- | --- |
| static MatrixWithHeaders [] broad.pda.differentialExpression.Permutations.permuteData | ( | MatrixWithHeaders | *data*, |
|  |  | Collection< String > | *group1*, |
|  |  | Collection< String > | *group2*, |
|  |  | int | *numberPermutations*, |
|  |  | double[] | *fudgeFactors*, |
|  |  | boolean | *useFold*, |
|  |  | boolean | *paired* |
|  | ) |  |  |

static

Here is the call graph for this function:

Here is the caller graph for this function:

|  |  |  |  |
| --- | --- | --- | --- |
| static MatrixWithHeaders [] broad.pda.differentialExpression.Permutations.permuteData | ( | MatrixWithHeaders | *data*, |
|  |  | Map< String, Collection< String >> | *groups*, |
|  |  | int | *numberPermutations*, |
|  |  | double[] | *fudgeFactors*, |
|  |  | boolean | *useFold*, |
|  |  | boolean | *paired* |
|  | ) |  |  |

static

Here is the call graph for this function:

|  |  |  |  |
| --- | --- | --- | --- |
| static MatrixWithHeaders [] broad.pda.differentialExpression.Permutations.permuteDataByGroupSize | ( | MatrixWithHeaders | *data*, |
|  |  | int | *groupSize*, |
|  |  | Map< String, Collection< String >> | *groups*, |
|  |  | int | *numPerm*, |
|  |  | double[] | *fudgeFactors*, |
|  |  | boolean | *useFold* |
|  | ) |  |  |

static

---

The documentation for this class was generated from the following file:

- java/broad/pda/differentialExpression/Permutations.java


---

Generated on Fri Aug 15 2014 15:10:59 for ESAT by  

 1.8.7
